# Application of Modern Research Methods for the Physicochemical Characterization of Ion Exchangers

**DOI:** 10.3390/ma14227067

**Published:** 2021-11-21

**Authors:** Yi-Gong Chen, Weronika Sofińska-Chmiel, Gui-Yuan Lv, Dorota Kołodyńska, Su-Hong Chen

**Affiliations:** 1 Collaborative Innovation Center of Yangtze River Delta Region Green Pharmaceuticals, Zhejiang University of Technology, Chaowang Road 18, Hangzhou 310014, China; yigongchen@hotmail.com; 2 Analytical Laboratory, Institute of Chemical Sciences, Faculty of Chemistry, Maria Curie-Skłodowska University, M. Curie Skłodowska Sq. 2, 20-031 Lublin, Poland; weronika.sofinska-chmiel@mail.umcs.pl; 3 College of Pharmaceutical Science, Zhejiang Chinese Medical University, Hangzhou 310053, China; zjtcmlgy@163.com; 4 Department of Inorganic Chemistry, Institute of Chemical Sciences, Faculty of Chemistry, Maria Curie-Skłodowska University, M. Curie Skłodowska Sq. 2, 20-031 Lublin, Poland

**Keywords:** ion exchangers, heavy metals, multifunctionality, optical profilometry, X-ray photoelectron spectroscopy

## Abstract

Ion exchange technique as the reversible exchange of ions between the substrate and the surrounding medium can be an effective way of removing traces of ion impurities from the waters and wastewaters and obtaining a product of ultrapure quality. Therefore, it can be used in analytical chemistry, hydrometallurgy, purification and separation of metal ions, radioisotopes and organic compounds, and it also finds great application in water treatment and pollution control. In the presented paper, the new trends for ion exchanger characteristics determination and application are presented. Special attention is paid to the ion exchangers with multifunctionality for heavy metal ions removal. They show superior actions such as sorption capacity values with excellent resistance to fouling and the possibility of application in the co-current or modern packed bed counter-current systems, as well as for the condensate polishing or the conventional mixed bed systems in combination with other resins. The results of the paper are expected to help researchers to establish a powerful strategy to find a suitable ion exchanger for heavy metal ions removal from waters and wastewaters. It is important because the best ion exchangers are selected for a specific application during laboratory tests taking into account the composition of the feed solution, pH, type of ion exchangers and then the column breakthrough tests. Therefore, the optical profilometry and the X-ray photoelectron spectroscopy can prove beneficial for this purpose in the case of three different ion exchangers such as Dowex M 4195, Amberlite IRA 743 and Purolite Arsen X^np^.

## 1. Introduction

Ion exchangers used in the ion exchange processes (IX) are the cross-linked/three dimensional materials forming the network called the matrix with fixed charges. The electro neutrality of the matrix is due to an equal number of ions of the opposite sign called counter-ions. They are free to move within the matrix and can be replaced by the other ions of the same sign when the ion exchanger is in contact with an electrolyte solution. Therefore, IX is applied in a large variety of industries including water treatment, food and beverage, pharmaceuticals, petrochemicals production as well as hydrometallurgy. It can be in a more privileged position especially for hydrometallurgical processes, because the extraction is characterized by larger valuable metal losses and smaller efficiencies [1,2,3,4,5,6,7,8].

### 1.1. Manufacture of Ion Exchangers and Main Processes of Production

During over sixty or seventy years of commercial production, the ion exchangers called Amberlite with the trademark of the Rohm & Haas Company, Philadelphia, Penn., USA, Diaion, with the trademark of Mitsubishi Corporation (previously Diamond Shamrock Corporation, Pittsburgh, Penn., USA), Dowex with the trademark of Dow Chemical Company (previously Dia-Prosin, France and Rohm & Haas, Chauny, France), Duolite, with the trademark of Dow Chemical Company, Imac with the trademark of Dow Chemical Company, Ionac/Relite with the trademark of the Sybron Corporation, Kastel as a trademark of Dow Chemical Company (previously Montedison, Milan, Italy), Lewatit, with the trademark Lanxess (previously Bayer, Leverkusen, Germany) as well as Purolite, with the trademark of Purolite International, and Resintech as a trademark of Resintech, Inc. were and are still used [9,10,11].

**Figure 1:**
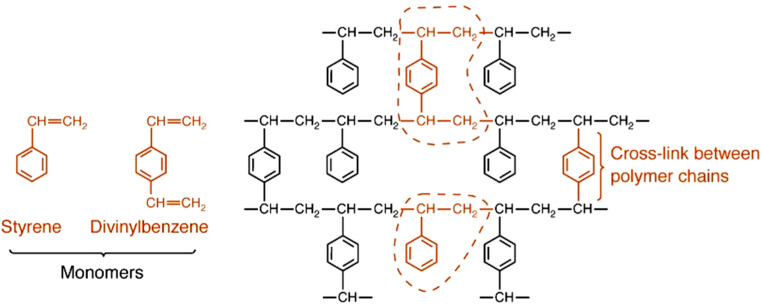
Synthesis of ST-DVB ion exchanger.

They are obtained from the two principal reactions, namely polycondensation and polymerization of monomers such as phenol and formaldehyde, styrene and divinylbenzene, acrylonitrile, acrylates and polyamines [12,13,14,15,16,17], leading to the cross-linked ion exchangers with a typical 4 to 20% cross-liking agent (mostly DVB) (Figure 1) [15].

### 1.2. Microporous, Macroporous and Hypercross-Linked as Well as Polyhydrophilic Ion Exchangers

**Figure 2:**
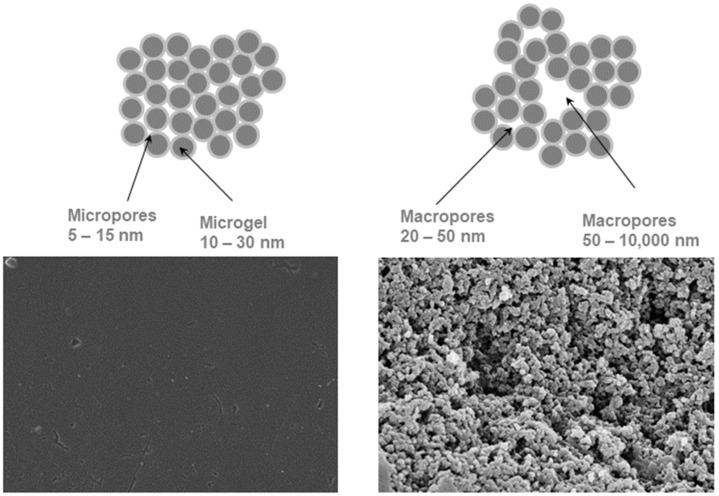
Gel (microporous) and macroporous ion exchangers.

The polymerization of only two monomers leads to the ion exchangers of the so-called gel (microporous) type, while the addition of an appropriate inert compound to the mixture of co-monomers leads to the ion exchangers of the porous (macroporous) type (Figure 2).

Macroporous ion exchangers, also known as the second generation polymer network, have a sponge-like structure that provides greater stress tolerance and resistance to fouling and physical breakdown (osmotic shocks, changes of pH, polarity), oxidizing reactions and swelling.

The third generation of polymer network is known as the hypercross-linked polystyrene (HCPs) ion exchangers [18,19,20]. The most common are Purolite (Hypersol-Macronet, MN-series) and Dow Chemical (Optipore) as well as Lanxess (Lewatit VP OC 1163 or S 7768). They are characterized by the large specific surface areas 600–2000 m^2^/g, permanent small pores and large micropore volumes. The sorption properties from the unfunctionalized hypercross-linked polystyrene with the high surface area above 2100 m^2^/g are mostly based on hydrophobic and π-π interactions [21]. However, their functionalization could be achieved by the post-polymerization chemical modifications or introducing a functional group containing monomers.

Ion exchangers with interconnected pores with a large content of HIPE (high internal phase emulsion) are known as polyhydrophilic polymers. They have a porosity of up to 90%, a low density of 0.1–0.2 g/cm^3^, a pore size in the wide range of 5–100 μm and a specific surface of up to over 500 m^2^/g. Therefore, they can be used in 3D cell culture tissue engineering, peptide synthesis, production of hydrogen carriers as well as modern catalysts and reagents designing [22].

### 1.3. Forms of Ion Exchangers

**Figure 3:**
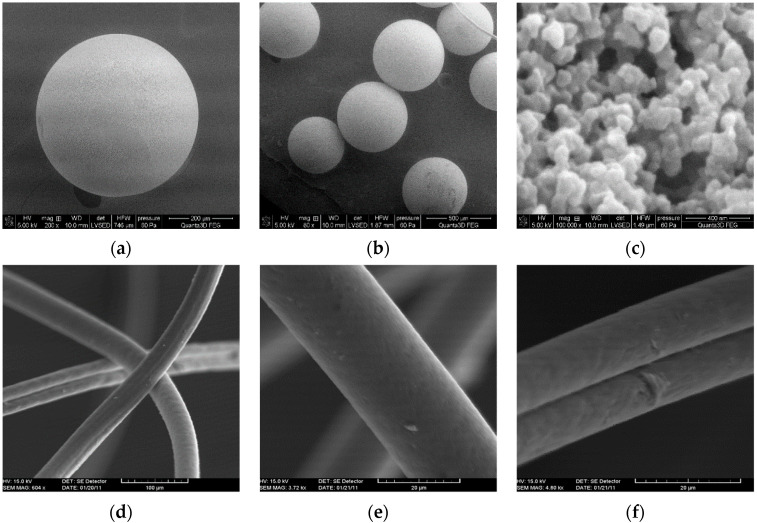
SEM images of Lewatit SP112 (**a**–**c**) and Fiban-X (**d**–**f**).

For industrial purposes, the ion exchangers can be obtained in the form of beads (IEB) or fibres (IEF) (Figure 3a–f). In the purification industry, where ion exchangers are used in the form of beads, the mesh size is often used to determine the particle size distribution. However, fibres can simultaneously work as mechanical filters and ion exchangers. They are mostly promising for continuous counter flow process technologies due to a more developed surface than beads, thus providing higher ion exchange rates [23]. As examples of fibrous ion exchangers, FIBANs (Figure 3d–f) (from Physical and Organic Chemistry Institute, Minsk, Belarus) and Smopex^®^ (from Johnson Matthey, London, UK) should be mentioned. Now, IEF are manufactured by Ecofil-Deco Company, Minsk, Belarus).

These types of ion exchangers are used, among others, for deep air purification from ammonia. These materials are characterized by the chemical properties comparable to those of the ion exchange resins such as mechanical strength and flexibility [24,25]. Small sizes of their monofibres (5–50 µm) and thickness uniformity allow for high speed and efficiency of the ion exchange process. They are also characterized by an extremely great rate of ion exchange, high osmotic stability allowing for their repeated drying and wetting as well as transformation from one ionic form into another without damaging the fibres [26,27]. The typical half-life of ion exchange processes for fibres is 2–10 s, while for the traditional ion exchangers it is 30–200 s. High process speed is achievable due to the short diffusion path. As a result, they can be used in the form of a thin filter layer of 7–20 mm at a high solution or gas flow rate for cleaning exhaust gases (from SO_2_, HCl, NO_x_, HF), removal of fumes (NH_3_, RNH_2_), odorant (RSH, RNH_2_) or air purification from tobacco smoke, e.g., in smokers’ cabins.

### 1.4. Types of Functional Groups

In the case of ion exchangers, the matrix can be a polyanion (negatively charged) used as a cation exchanger or a polycation (positively charged) used as an anion exchanger. To convert the ST-DVB matrix to an ion exchanger, various functional groups can be attached. The most common functional groups are sulphonic (strongly acidic cation exchangers, SAC), quaternary ammonium of types 1, 2 or 3 (strongly basic anion exchangers, SBA), carboxylic (weakly acidic cation WAC exchangers), amine, polyamine (weakly basic anion WBA exchangers,) and chelating with the iminodiacetic, phosphinic, phosphoric, aminophosphonic, thiol, thiourea, isothiouronium, amidoxime, etc., units [28]. However, among the chelating ion exchangers, those with the iminodiacetic and aminophosphonic groups predominate [29,30].

**Figure 4:**
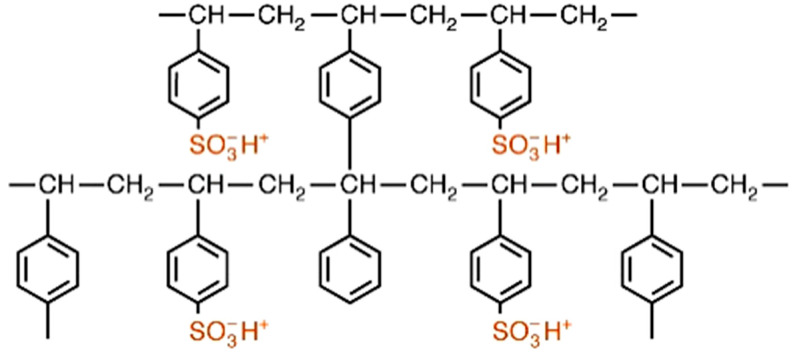
Structure of cation exchanger with the sulfone functional groups.

Some of the above-mentioned ion exchangers are characterized as below:

✓ Strongly acidic cation (SAC) exchangers possess good physical and oxidation stability, variable capacities (5.4 meq/L) depending on the regeneration levels, but small operating efficiency (in the case of water softening in the Na^+^ cycle and demineralization) (Figure 4).

**Figure 5:**
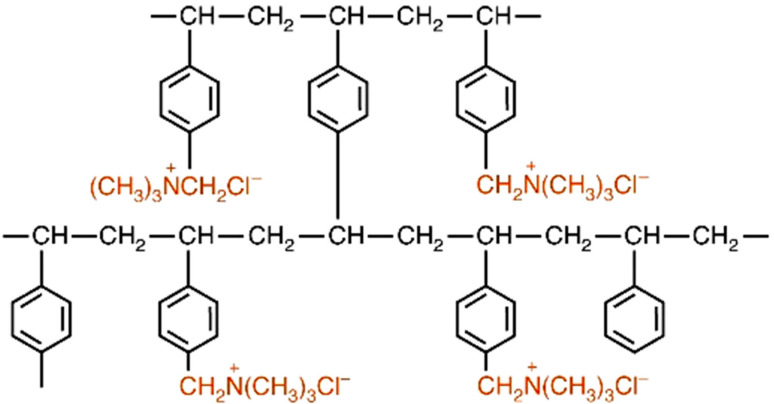
Structure of anion exchanger of type 1.

✓ Weakly acidic cation (WAC) exchangers with a partial exchange capacity, much higher capacity than SAC, high operating efficiency, but the limitations include a relatively high cost, poor kinetics and physical stability (for dealkalization in the H^+^ cycle and for brackish water softening in the Na^+^ cycle), weak acid groups such as -COO^−^, ionise only at high pH. At low pH, that is, a high concentration of hydrogen ions, they form undissociated -COOH [31].
✓ Strongly basic anion exchangers (SBA) with a quaternary ammonium group with good kinetics and short rinses, but there is a trade-off between the exchange efficiency and the resulting quality. They are thermodynamically unstable, prone to organic fouling and cost considerably more than the cation equivalent. Their capacities are equal to 2.5–4.0 meq/g. The SBA ion exchangers of type 1 (trimethylammonium) and type 2 (dimethylhydroxyethylammonium) quaternary ammonium functional anion exchangers are obtained by amination of the polymer matrix with trimethylamine (TMA) or dimethylethanolamine (DMEA). Type 1 ion exchangers are either gel with a particularly suitable temperature and oxidation stability or macroporous with higher capacity and resistance against organic fouling (Figure 5).

They are used for treating waters and wastewaters with a small concentration of free mineral acids, with a large of silica and where a small silica leakage is required. The higher cost of the type 1 ion exchangers is considered acceptable in order to obtain a more efficient and longer-lasting resin. Type 2 ion exchangers provide much better capacity, excellent regeneration efficiency and resistance to fouling; however, they are limited to the lower temperature operation < 35 °C with a larger SiO_2_ leakage. Therefore, they are used in small plants. Type 3 ion exchangers with the tri-n-butyl ammonium functional groups are designed to offer the highest selectivity for trace contaminants such as nitrate or perchlorate.

✓ Weakly basic anion (WBA) exchangers with a tertiary amine group removes only strong (hydrochloric, sulfuric, nitric) acids. They are characterized by a large operating capacity and regeneration efficiency with good resistance to organic fouling and thermal stability, but longer rinse times, poor kinetics and they cost even more than SBA. Weak base groups lose a proton when the pH value is high, forming uncharged NH_2_ ions. Consequently, for the resins which are weakly ionic, the exploitable capacity depends on the pH of the liquid being treated. Weak base anion exchangers are particularly effective in handling natural organics [32].

### 1.5. Affinity of Ion Exchangers

It is well known that the affinity of sulfonic acid resins for cations varies with the ionic size and charge of the cation. Generally, the affinity is greater for large ions with high valency. For dilute solutions the order of affinity for some common cations is approximately:

Hg^2+^ < Li^+^ < H^+^ < Na^+^ < K^+^ ≈ NH_4_^+^ < Cd^2+^ < Cs^+^ < Ag^+^ < Mn^2+^ < Mg^2+^ < Zn^2+^ < Cu^2+^ < Ni^2+^ < Co^2+^ < Ca^2+^ < Sr^2+^ < Pb^2+^ < Al^3+^ < Fe^3+^.

A corresponding list for the amine based anion exchangers is: OH^−^ ≈ F^−^ < HCO_3_^−^ < Cl^−^ < Br^−^ < NO_3_^−^ < HSO_4_^−^ < PO_4_^3−^ < CrO_4_^2−^ < SO_4_^2−^.

The temperature, degree of ionisation of solvent, solute and ion exchanger are important for the ion exchange capacities [33,34].

### 1.6. Ion Exchangers Advantages and Applications

The ion exchange process similar to adsorption (with the contribution of ions) plays an important role in the water treatment to enable recycling of such waters and reduces the demand for freshwater resources. This is still the only practicable option with full and part flow for treatment of ammoniated and microbiological wastewaters [35,36,37,38,39,40,41]. The main advantages of ion exchange are the recovery of valuable substances, selectivity, reduced sludge formation and compliance with the discharge specifications compared to the other methods such as chemical precipitation, adsorption, membrane filtration, coagulation–flocculation and floatation as the materials belonging to active charcoals, zeolites, hybrid materials, MOFs, etc. [42,43,44].

Among them, of significant importance are those connected with effective sorption of metal ions from acidic solutions with the complex matrix, selective sorption of the specific group of metal ions such as alkali, alkali earth, heavy metal or lanthanide ions. In many cases with high sorption capacity, several sorption/desorption cycles and not large ecological footprint are found [45,46,47,48,49,50,51,52,53]. Ion exchangers in the weakly acidic forms are also useful in food production, proteins purification, colour removal from cane sugar syrups to make white sugar as well as to purify antibiotics and other pharmaceuticals [54,55,56,57].

The resins are also used as active ingredients in drug formulations for pills disintegration, taste-masking, controlled or extended-release, stabilization as well as for drug isolation or purification. The drug-resin complex can be used in the suspension or isolated as a dry, solid, free-flowing, stable compound. The drug is released from the resinate upon exposure to physiological fluids [45,46].

Physical characterization of ion exchangers can be made by means of different methods. In the presented paper the optical profilometry and the X-ray photoelectron spectroscopy were used to characterize the commercially available ion exchangers Dowex M 4195, Amberlite IRA 743 and Purolite Arsen X^np^ before and after the sorption of Cu(II) or As(V). These two methods are rarely used together to characterize ion exchangers. Finding the relation between the compositional differences, topography, shape, inclination, edges, or physical differences, etc., and the type of elements, their oxidation state and binding energy is very useful for prediction of mechanism sorption of different adsorbents. In order to examine the internal structure, the beads were cut using an ultramicrotome and then imaged, e.g., using a high resolution scanning electron microscope. For describing the distribution of elements before and after the sorption process the profiles were registered and the roughness parameters were determined. This type of research on microgeometry and roughness analysis of cut beads of the Dowex M 4195, Amberlite IRA 743 and Purolite Arsen X^np^ ion exchangers were not described in the literature.

## 2. Materials and Methods

### 2.1. Ion Exchangers

The physicochemical characteristics of Dowex M 4195, Amberlite IRA 743 and Purolite Arsen X^np^ are presented in Table 1.

The sorption tests were carried out using Cu(II) or As(V) solutions. The solutions of Cu(II) and As(V) were prepared for experiments by dissolving CuCl_2_·6H_2_O or Na_2_HAsO_4_·7H_2_O in water. The specific pH of the solutions was achieved by adding appropriate amounts of 1 M HCl and 1 M NaOH. To test sorptive properties of Dowex M 4195, Amberlite IRA 743 and Purolite Arsen X^np^ forwards Cu(II) or As(V), respectively, 20 mL solutions of 100 mg/L concentration at pH 2.0 or 6.0 and 0.1 g of the selected ion exchanger were placed in 100 mL conical flasks and shaken using laboratory shaker ELPIN+ type 358A (Lubawa, Poland) in the time of 6 h. The above experiments were carried out at room temperature with a shaking speed of 180 rpm and amplitude 7. All samples after shaking were separated by filtration using the filter paper. The concentration of Cu(II) ions was determined by means of atomic absorption spectrometry (AAS) using the spectrometer SpectrAA240 FS (Varian Inc., Melbourne, Australia). The concentrations of arsenic(V) ions were determined using the UV-Vis method (Cary 60, Agilent Technologies, Santa Clara, CA, USA).

### 2.2. Instruments

Optical profilometry enables precise, spatial microgeometry imaging of the surface of selected ion exchangers Dowex M 4195, Amberlite IRA 743 and Purolite Arsen X^np^. The basis of the profilometer operation is the phenomenon of light interference and analysis of the interference fringes pattern. The light beam, whose source is the LED, is divided by passing through the interference lens. One part of the beam is reflected from the sample surface, the other from the reference mirror. Returning, both beams interfere with each other, creating an interference pattern on the detector of the surface being tested. The location of the obtained profile in the axis Z gives the possibility to generate a 3D map of the studied area (the resolution in the Z axis is independent of the scanning range and being equal to 3 nm). The profilometer ContourGT-K1 (Vecco, Bruker, Leiderdorp, The Netherland) works in two ways: VSI (Vertical Scanning Interferometry) with the maximum height of scaning 10 mm) and PSI (Phase Shifting Interferometer) for super smooth surfaces with less than 30 nm roughness. Before performing profilometric tests, the ion exchanger beads were cut with an ultramicrotome. The step in the ultramicroton was set to 5 nm. The tests were performed using an accredited method for three ion exchanger beads before and after the sorption process. To this aim, the software Vision 64, ver. 5.41 update 4 was applied. According to the test procedure, five measurements are made for each object and the result is given as the average of five measurements. In the accredited method, the measurement uncertainty was estimated: The roughness measurement uncertainty, R_a_, was estimated, taking into account repeatability, recovery and decalibration of the apparatus and the uncertainty of the standard. The uncertainty was estimated for the two extreme points of the R_a_ measurement range (upper and lower limits) with the assumption of a trapezoidal distribution. The expanded uncertainty of the roughness measurement was given with and without taking into account the standard uncertainty.

**Figure 6:**
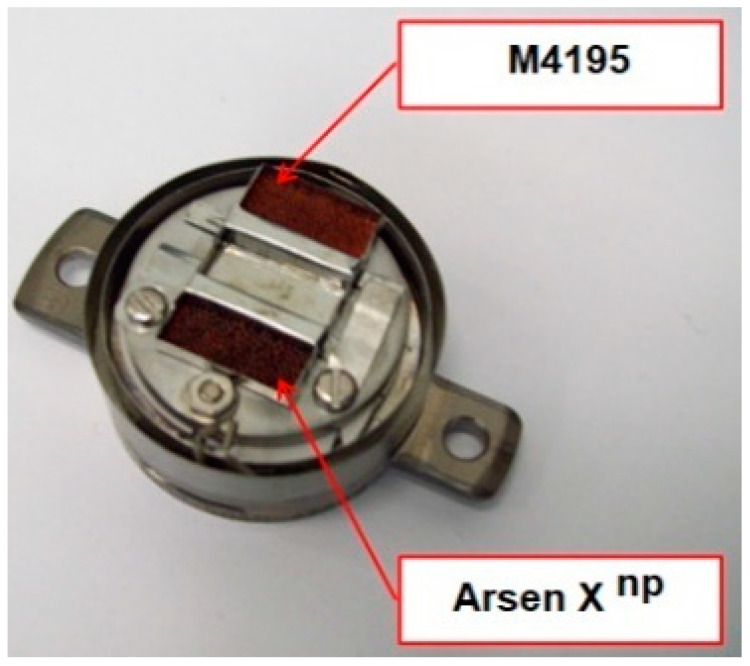
Samples mounted on a carrier.

In order to investigate the chemical structure of the ion exchangers, the studies were carried out by means of the ultra-high vacuum multi-chamber analytical system (Prevac, Rogowo, Poland). After mounting on a molybdenum carrier (Figure 6), the samples were degassed at room temperature to a constant high vacuum of ~5 × 10^−8^ mbar, in the loading sluice of the UHV system.

AlKα monochromatic radiation was used as a source of photoelectrons. Photoelectrons were stimulated by X-ray of a characteristic line AlKα of 1486.7 eV energy, generated by a VG Scienta SAX 100 lamp with an aluminium anode with a VG Scienta XM 780 monochromator. The pressure in the chamber during the measurements was 2 × 10^−8^ mbar. The X-ray tube operating parameters were as follows U 12 kV, Ie 30 mA.

Additionally, the morphology of the ion exchangers before and after the Cu(II) or As(V) sorption was determined using scanning electron microscopy (SEM) (Quanta 3D, FEG, Hillsboro, OR, USA). 907 Titrando set (Metrohm, Herisau, Switzerland) was applied to determine pH_pzc_ by means of the pH potentiometric titration method.

Using an ASAP 2405 analyser (Micromeritics Instrument Corporation, Norcross, GA, USA) porous structure parameters of the ion exchangers were evaluated using the Brunauer–Emmett–Teller and Barret–Joyner–Halenda models. These results are presented in Table 1.

## 3. Results and Discussion

The physicochemical characteristic of Dowex M 4195, Amberlite IRA 743 and Purolite Arsen X^np^ are presented in Table 1 and Table 2. The SEM images for Dowex M 4195 and Purolite Aren X^np^ were obtained for 10,000× magnification and Amberlite IRA 743 1000×.

These ion exchangers are of ST-DVB macroporous type. The bead size is also of significant importance. It is well known that loading rates decrease as the resin bead sizes increase so that a smaller resin bead will load much quicker than a large one. For the analysed ion exchangers, it changes in the range: 0.300–0.8500 mm, 0.500–0.700 mm and 0.300–1.200 mm, respectively. Based on Table 1 and Table 2, it can be stated that the macroporous resins are opaque and have a permanent porosity even in the dry state. Generally, they are characterized by the smaller capacity, surface area in the range 20–300 m^2^/g (in our case 3.2–14.5 m^2^/g), total pore volume of about 1 cm^3^/g and an average pore diameter of about 20 nm.

Contrary to the gel ion exchangers, they have a higher degree and nonuniform cross-linking which decreases kinetics, capacity and operating efficiencies, but maximizes strength and resistance to fouling. In the solvated state, the ion exchanger beads contain mainly macropores. The pore dimension depends on the swelling degree. In the dry state, the resins are characterized by high porosity and the beads are not translucid [58]. The small particles associate in clusters, forming macropores. The clusters aggregate and the voids form the third family of macropores with the diameters of 50–1000 nm. Although the majority of the internal pores are mesopores according to IUPAC classification, they are called macroporous (in the case of Amberlites ion exchangers they are called macroreticular) [59]. Particularly, Dowex M 4195 is regarded as a material intermediate between the ion exchanger and the non-porous one that is with a not completely functionalized matrix [60,61,62,63,64]. As was mentioned in [63], such resin is characterized by a large operating capacity (higher regeneration efficiency), smaller leakage, and greater physical and chemical stability than the conventional and fully functionalized resins of the same particle size. Within the structure, the mechanically stable core and film of the bispicolylamine functional groups can be distinguished. Dowex M 4195 was used to control the Ni(II) levels from 0.80–0.90 g/dm^3^ to below 0.16 g/dm^3^ in the Co(II) electrolyte (40 g/dm^3^) in the INCO, Port Coulbourne, Ontario, Canada and ENRC’s Chambishi Metals in Zambia, Africa [59]. Moreover, Metals Finance Africa used it for the recovery and purification of nickel sulphate from the Cu(II) electrolyte in Rio Tinto’s Palabora Mining Company, Phalaborwa, South Africa.

### 3.1. Optical Profilometry of Ion Exchangers

The surface texture, especially its roughness and waviness, is characterized by many parameters, among which the amplitude-height parameters are the most important. In the case of Dowex M 4195, Amberlite IRA 743 and Purolite Arsen X^np^, these parameters were determined before and after the sorption of metal ions.

The most useful for the assessment of surface roughness of ion exchangers are the following parameters: R_a_, the arithmetic mean of the elevation profile, R_q_, the mean square elevation profile, and maximum profile height R_t_. The roughness measurement uncertainty, R_a_ was estimated taking into account repeatability, recovery, decalibration of the apparatus and pattern uncertainty. The uncertainty was estimated for the two extreme points of the R_a_ measurement range (upper and lower limits) with the assumption of a trapezoidal distribution. This kind of analysis is very useful for effective sorption characterization and can be applied in the dynamic method.

The examples of the surface microgeometry analysis for the cut beads of Dowex M 4195 ion exchanger before and after the Cu(II) ion sorption are presented in Table 3 (due to the two-layer structure of Dowex M 4195, the maps were made of the middle and edge parts of the cut beads).

Analogous tests were carried out for the Amberlite IRA 743 ion exchangers before and after the Cu(II) sorption (Table 4).

The profilometric tests were also carried out for the cut beads using ultramicrotome for Purolite Arsen X^np^ before and after the As(V) ion sorption (Table 5).

The surface microgeometry maps from the edge and middle parts of the beads are shown in Table 3, Table 4 and Table 5. The tests carried out using an optical profilometer allowed the determination of the following roughness parameters: arithmetic mean of profile ordinates (R_a_), square mean of profile ordinates (R_q_) and maximum profile height (R_t_).

It was found that Amberlite IRA 743 shows the highest values of R_a_ and R_q_ compared to the other ion exchangers under discussion before and after the sorption process. After the sorption of Cu(II) ions, the R_a_ values increase from 203 to 550 nm for Dowex M 4195, from 771.44 to 751.34 nm for Amberlite IRA 743 and decrease from 770.49 to 627.51 nm for Purolite Arsen X^np^. The analogous tendency was observed for the *R*_q_ parameter. The largest differences were observed for Dowex M4195. The studies have also shown the differences in the edge middle roughness of the ion exchangers. The largest differences were observed in the Dowex M4195 ion exchanger, which is characterized by a two-layer structure. What is interesting a quite similar structure compared to Dowex M 4195 can be observed in the case of shallow shell resins (SST) or pellicular ion exchangers (IEP) with the glass or silica core. IEPs are mechanically strong and can cope with high pressures very easily. Due to the fact that the amount of ion exchange material on the core is very small, they are characterized by low capacity and can be easily overloaded, although they can produce very fast separations [65]. The fast ion exchange rate favours a sharp boundary in the column between the two zones formed by the exhausted resin and that in the initial ionic form, increasing the degree of column utilization. The SST cation resins reduce effectively fouling and leakage from Ca(II), Mg(II), Fe(III), Ba(II) and Sr(II), while the SST anion resins minimize greatly organic fouling and silica leakage. The technology is compatible with co-flow, counter-flow and packed bed systems, improving economic and process results for softening and demineralization applications. In this group, Purolite Shallow Shell™ SSTPFA64, Purolite SST60H and Purolite SST80H are well-described.

Equally interesting is the ion exchanger Amberlte IRA 743, selective towards boron, similarly to the other ion exchangers used for boron removal such as Lewatit MK 51 and Purolite S 110 [66,67,68,69]. It contains the N-methylo-D-glucamine (NMDG) functional groups grafted on the ST-DVB skeleton and it is commonly used for the selective removal of boron in the form of trioxoboric acid or B(OH)_4_^−^ ions from the sea water and wastewaters. Due to the presence of -OH groups closely attached in the cis position in the N-methyl-D-glucamine functional group, complexes are formed with H_3_BO_3_ and its salts. Regeneration is affected by HCl or H_2_SO_4_, lime or ammonia. This resin can be used for post-treatment desalination or production of ultrapure water. The efficiency of Amberlite IRA 743 resin is independent of temperature, pH and salinity.

The group of the hybrid ion exchangers (HIXs) includes Purolite Arsen X^np^, also known as FerriIX™ A33E—a strongly acidic macroporous cation exchanger based on the ST-DVB skeleton with the attached sulfonic acid groups and containing hydrated iron oxide nanoparticles for sorption of not only oxyanions AsO_4_^3−^, but also HPO_4_^2−^, HSbO_4_^2−^, SCN^−^, etc. [70,71,72,73,74,75,76]. In this group, there is also Lewait FO36—hybrid anion exchanger (HAIX) containing dispersed nanoparticles of hydrated ferric oxide (HFO) with Fe(III) and quaternary ammonium functional group (-^+^NR_3_) as well as Bayoxide^®^ E 33 [71,72]. They can be used for arsenic removal from potable water in which both arsenate As(V) and arsenite As(III) are safely adsorbed below 5 μg/dm^3^.

The hybrid polymeric–inorganic ion exchangers selective towards As(III,V) were introduced by Cumbal and SenGupta [73,74,75]. High concentration of the functional groups allowed large and fairly uniform loading of hydrated ferric oxide (HFO) particles (approximately 9–12% of Fe(III) by mass) within the polymeric beads. The additional advantage of such solution was simultaneous removal of not only As(III,V) ions, but also SO_4_^2−^, Cl^−^ and HCO_3_^−^. As opposed to As(III,V) these competitive ions form only outer sphere complexes with only weak affinities from HFO and they are characterized by smaller selectivity. The regeneration process can be achieved using the NaOH solution [76].

Beside incorporation of HFO into the structure of a cylindrical polymeric ion exchange fibre, hybrid sorbents with the capacity up to 15 mg/g were obtained [77]. In this way effective sorbents based on Amberlite PWA2, Purolite A-530E, SIR-110, CalRes 2103, Purolite A-520E and SIR-100 for chlorates(VII) removal can also prepared [77]. Pan et al. [78] proposed a novel process to immobilize the nanoparticulate HFO within the macroporous anion exchange resin D-201 for phosphate removal from aqueous systems.

Taking the manufacturers information into account the ratio of elements, which are present on the ion exchanger surface to into ion exchanger matrix, should be the highest in the case of Cu(II) for Dowex M 4195, B(III) for Amberlite IRA 743 or Fe(III) and As(V) for Purolite Arsen X^np^. It is evident that in the case of Dowex M4195, the above-mentioned ions are on the surface of the ion exchanger and do not diffuse into the beads [60]. Using the adsorption Langmuir model, the parameters of the maximum surface coverage, i.e., the maximum monolayer capacity for studied ion exchangers towards copper (II) increased in the following series: Dowex M 4195 (50.38 mg/g) > Amberlite IRA 743 (45.13 mg/g). For Purolite Arsen X^np^, the maximum sorption capacity towards arsenic(V) was equal to 22.37 mg/g (pH 6, shaking speed 180 rpm, temperature 295 K) [60,61].

### 3.2. X-ray Photoelectron Spectroscopy (XPS) of Ion Exchangers

There are many research techniques used to obtain information about the physicochemical properties of ion exchangers and sorbents after the sorption process. The photoelectron spectroscopy can be one of them. This method measures distribution of the electrons energy emitted from the sample which absorbed ultraviolet photons (ultraviolet photoelectron spectroscopy, UPS) or X-rays radiation (X-ray photoelectron spectroscopy, XPS). The literature reports numerous data resulting from the analysis of different types of ion exchangers using the XPS method.

In the paper, the photoelectrons were recorded by the hemispherical analyser Scienta R4000. The measurements were made based on the following basic parameters: operating mode—sweeping, pass energy 200 eV, measured range of the binding energy of photoelectrons 0–1200 eV, measuring step 0.5 eV, collection time in a single step 0.2 s and the number of iterations was 5. There were applied the following parameters of the analyser for the high resolution spectra: operating mode sweeping, pass energy 50 eV, measuring step 0.1 eV and collection time in a single step 0.667 s. The CasaXPS software was used to process the XPS spectra and calculate the quantitative surface composition of the tested materials. The review spectra were used to calculate the quantitative surface composition of the tested materials. For the purposes of chemical analysis, i.e., the assessment of functional groups, the XPS spectra recorded in a narrow range of energy were used.

**Figure 7:**
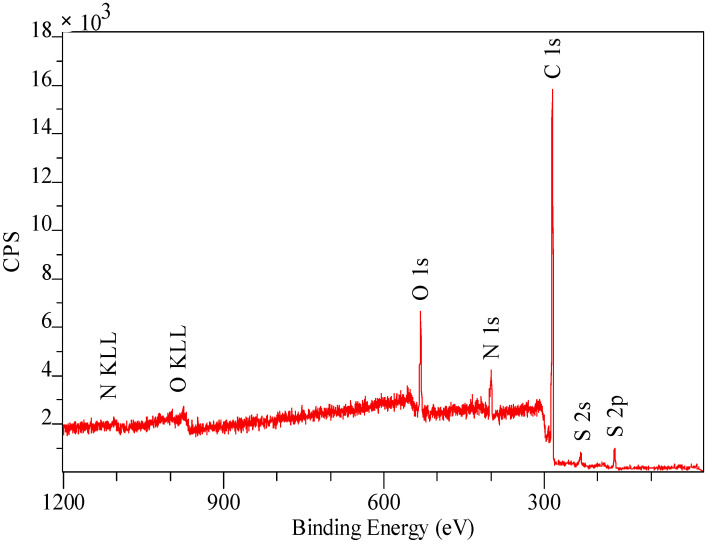
XPS spectrum obtained in a wide range of binding energy made for the Dowex M 4195 before the sorption process.

**Figure 8:**
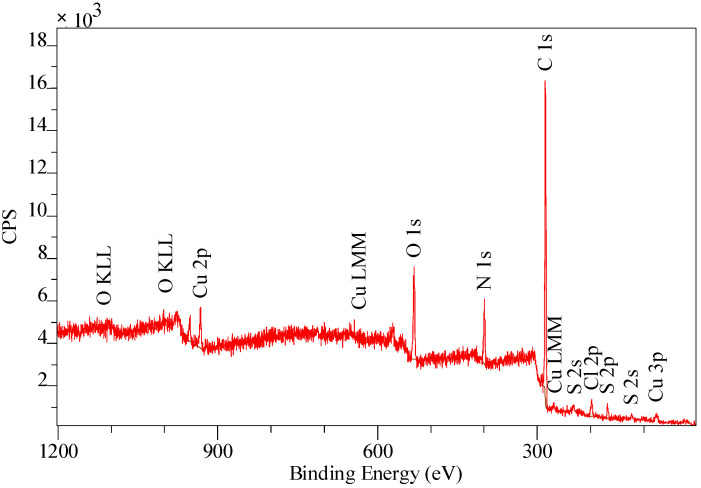
XPS spectrum obtained in a wide range of binding energy made for the Dowex M 4195 after the Cu(II) sorption.

**Figure 9:**
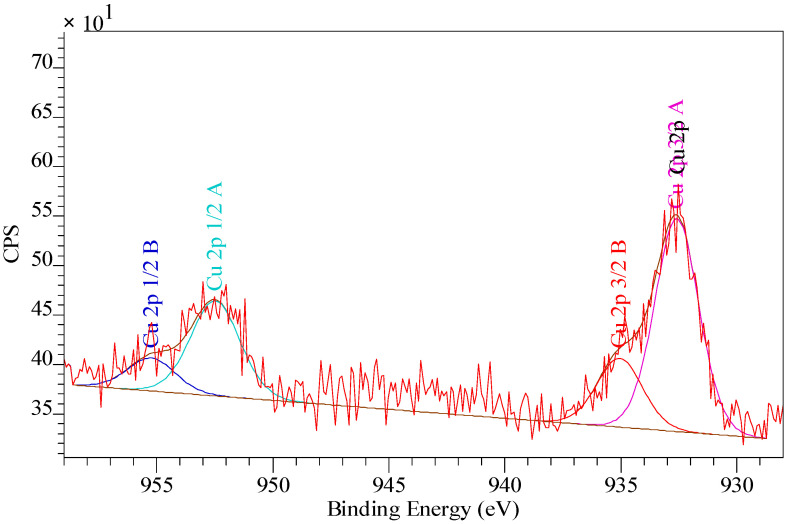
XPS spectrum obtained in a narrow range of binding energy made for Dowex M 4195 after the Cu(II) sorption.

Due to the fact that the XPS bands are characterized by small resolution, they were “decomposed” (deconvolution method) using the Gauss/Lorentz asymmetric function. To normalize the spectroscopic measurements, the X axis of the spectra (binding energy) was calibrated to a C1s aliphatic carbon peak, EB = 285 eV. The exemplary results are presented in Table 6, Table 7, Table 8 and Table 9 and Figure 7, Figure 8 and Figure 9. Analogous results can be obtained for Amberlite IRA 743 and Purolite Arsen X^np^.

The XPS tests showed the presence of the following elements: 82.2% carbon 7.9% oxygen, 7.5% nitrogen and 2.4% sulphur (Table 6). Of the total number of carbon atoms, 66.6% is in the form of C = C, C-C, C-H. 68.9% of oxygen present on the surface in is in the form of SO_4_^2−^, the remaining oxygen is in the form of O-H groups (Table 7) [79]. The study showed the presence of two forms of nitrogen [80,81,82]. All nitrates present on the ion exchangers surface come from the functional groups. The ratio of nitrogen from the pyridine rings to the amine nitrogen is 2:1, which is consistent with the theoretical data provided by the ion exchanger manufacturer because in the bis-(2-pyridylmethyl)amino function group, one nitrogen in the form of an aliphatic amine accounts for two pyridine nitrogen atoms [83]. The XPS tests showed the presence of sulphur as SO_4_^2−^ (Table 7), which is also consistent with the data of the ion exchangers manufacturer [84]. The percentage of individual elements is given in atomic%. The XPS study of Dowex M4195 ion exchanger was also carried out after the Cu(II) ion sorption process (Table 8 and Table 9, Figure 8 and Figure 9).

**Figure 10:**
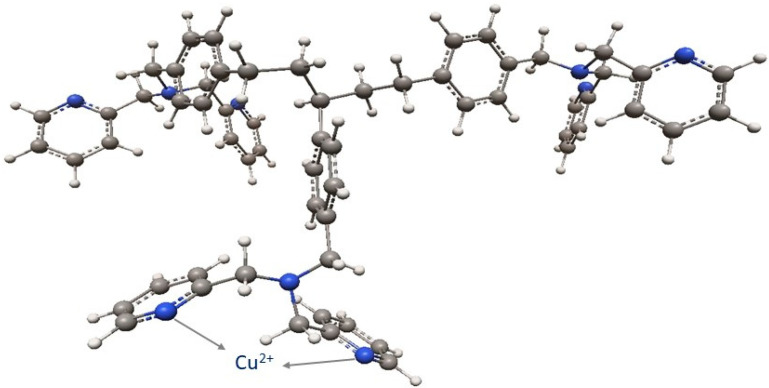
Cu(II) ions complexation by Dowex M 4195 proposed on the basis of theoretical calculations with the bis-(2-pyridylmethyl)amino groups attached in the *para* position.

The XPS tests carried out after the Cu(II) ion sorption on the Dowex M 4195 ion exchanger showed a shift of the band from nitrogen atoms in the pyridine ring towards higher binding energies, i.e., from 399.3 eV to 399.6 eV [84]. The XPS studies showed also the presence of three forms of copper: Cu(0), Cu(I) and Cu(II). This type of analysis is helpful for the sorption mechanism prediction. The research suggests that the main role in the formation of coordination bonds is played by the nitrogen atoms present in the pyridine ring which can be presented in Figure 10.

Therefore, in the case of Dowex M 4195 with the bis-(2-pyridylmethyl) amino) functional groups (BPA) proposed mechanism of Cu(II) ions sorption can be as follows:

R–N(BPA)_2_ + Cu^2+^ ⇄ R–N(BPA)_2_ ⋯ Cu^2+^

for Amberlite IRA 743 with the N-methylo-D-glucamine functional groups:

R–NMDG + Cu^2+^ ⇄ R–NMDG ⋯ Cu^2+^

and Purolite Arsen X^np^ with the sulfonic groups with hydrated iron oxide nanoparticles

R-SO_3_H/FeO(OH) + H_2_AsO_4_^−^ ⇄ [R-SO_3_H/FeO]H_2_AsO_4_ + OH^−^

2R-SO_3_H/FeO(OH) + HAsO_4_^2−^ ⇄ 2[R-SO_3_H/FeO]_2_HAsO_4_ + 2OH^−^

where R is the Dowex M 4195, Amberlite IRA 743 or Purolite Arsen X^np^ skeleton (PS-DVB), BPA are the bis-(2-pyridylmethyl) amino) functional groups, NMDG are the N-methylo-D-glucamine functional groups and -SO_3_H/FeO(OH) are the sulfonic groups with hydrated iron oxide nanoparticles.

## 4. Conclusions

In this paper, the new trends for ion exchangers characteristics and application were presented. Special attention was paid to the ion exchangers with multifunctionality for heavy metal ions removal Dowex M 4195, Amberlite IRA 743 and Purolite Arsen X^np^. The use of optical profilometry allowed for the physicochemical characterization of ion exchanger beads. The tests showed the two-layer structure of the Dowex M4195 ion exchanger and the homogeneous internal structure of Amberlite IRA 743 and Purolite Arsen X^np^. These studies allowed us to determine the roughness parameters of the ion exchangers cut with the ultramicroton. Information on the roughness of the ion exchanger in future studies will be related to the depth of penetration of the sorbed ions into the ion exchanger beads. Moreover, the microscopic tests carried out using optical microscopy allowed the observation of the two-layer structure of Dowex M 4195 ion exchanger which is very important taking into account its application. XPS spectroscopy turned out to be an excellent tool for studying changes in the area of an ion exchanger molecule after the sorption processes. This enables the quantitative determination of the sorbed metal content on the ion exchanger surface and other analytical techniques should be used to quantify the amount of sorbed metals in the total volume of beads. However, XPS spectroscopy is very effective for the observation of sorption mechanisms. The XPS studies showed that the process of Cu(II) ions sorption on Dowex M 4195 occurs mainly with the share of nitrogen atoms present in the pyridine rings originating from the bis-(2-pyridylmethyl) amino) functional groups, which was confirmed by the theoretical calculations. Therefore, the optical profilometry and the X-ray photoelectron spectroscopy can prove beneficial for this purpose. The proposed methods were also helpful for the Amberlite IRA 743 and Purolite Arsen X^np^ characterization.

## Figures and Tables

**Table 1:** Physicochemical properties of Dowex M 4195, Amberlite IRA 743 and Purolite Arsen X^np^.

Properties	Dowex M 4195	Amberlite IRA 743	Purolite Arsen X^np^
Appearance	dark green	white	reddish-brown
Matrix	ST-DVB	ST-DVB	ST-DVB
Structure	macroporous	macroporous	macroporous
Functional groups	bis-picolylamine, bis(2-pyridyl-methyl) amine	N-methyl- D-glucamine (free base form)	sulfonic acid groups/hydrated iron oxide nanoparticles
Size (mm)	0.300–0.8500	0.500–0.700	0.300–1.200
pH range	0–14	0–14	4–9
Max temp. (°C)	80	75	80
S_BET_ (m^2^/g)	3.2	14.5	7.1
pH_ZPC_	2.4	8.00	8.31
Total capacity (eq/L)	1.4 (6 g Cu(II)/L)	0.7	0.8

**Table 2:** SEM images of Dowex M 4195, Amberlite IRA 743 and Purolite Arsen X^np^.

Dowex M 4195	Amberlite IRA 743	Purolite Arsen X^np^
before the sorption of Cu(II) or As(V)		
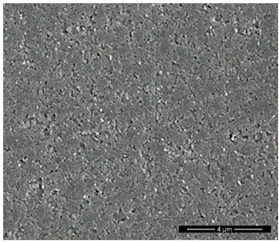	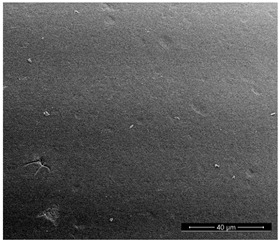	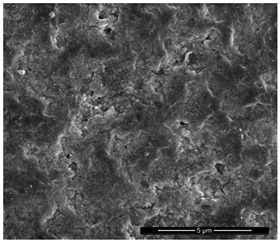
after the sorption of Cu(II) or As(V)		
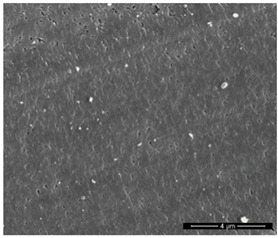	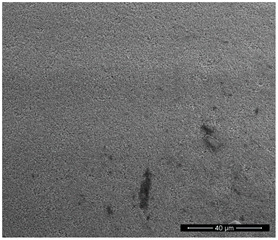	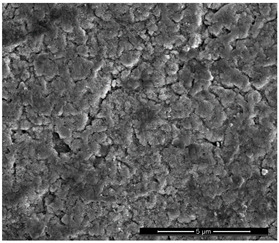

**Table 3:** Surface microgeometry and roughness parameters of Dowex M 4195 before and after the sorption of Cu(II).

Ion Exchanger	Surface Microgeometry Maps (Middle Part of the Cut Beads)	Surface Microgeometry Maps (Edge Part of the Cut Beads)
Dowex M4195 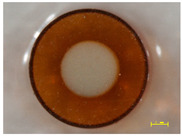	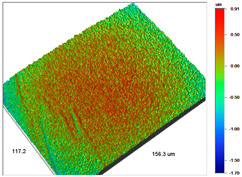	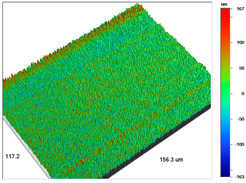
Roughness parameters	R_a_—203.86 nm ± 27.72 nm R_q_—254.39 nm R_t_—2.62 um	R_a_—24.41 nm ± 3.31 nm R_q_—31.10 nm R_t_—0.329 um
Dowex M4195-Cu(II) 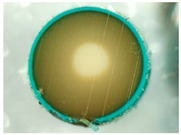	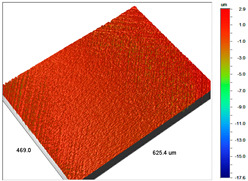	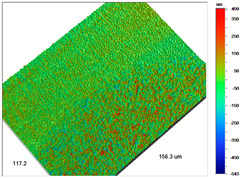
Roughness parameters	R_a_—550.13 nm ±74.81 nm R_q_—999.39 nm R_t_—20.53 um	R_a_—56.69 nm ± 7.71 nm R_q_—76.61 nm R_t_—0.952 um

**Table 4:** Surface microgeometry and roughness parameters of Amberlite IRA 743 before and after the sorption of Cu(II).

Ion Exchanger	Surface Microgeometry Maps (Middle Part of the Cut Beads)	Surface Microgeometry Maps (Edge Part of the Cut Beads)
Amberlite IRA 743 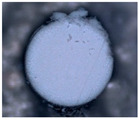	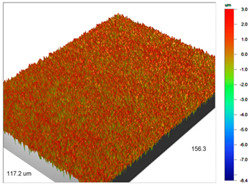	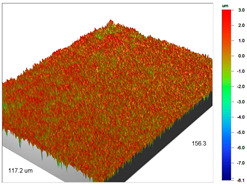
Roughness parameters	R_a_—771.44 nm ± 104.91 nm R_q_—986.66 nm R_t_—11.38 um	R_a_—799.77 nm ±108.78 nm R_q_—1020.00 nm R_t_—11.14 um
Amberlite IRA 743-Cu(II) 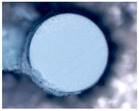	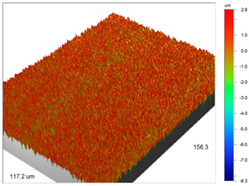	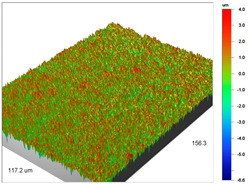
Roughness parameters	R_a_—751.34 nm ± 102.18 nm R_q_—959.35 nm R_t_—10.95 um	R_a_—698.34 nm ± 94.97 nm R_q_—895.70 nm R_t_—10.57 um

**Table 5:** Surface microgeometry and roughness parameters of Purolite Arsen X^np^ before and after the sorption of As(V).

Purolite Arsen X^np^ 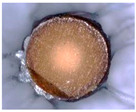	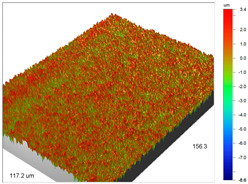	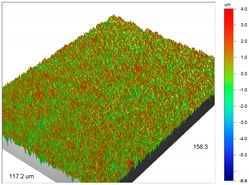
Roughness parameters	R_a_—770.49 nm ± 104.78 nm R_q_—985.56 nm R_t_—12.02 um	R_a_—698.25 nm ± 94.96 nm R_q_—895.70 nm R_t_—10.57 um
Purolite Arsen X^np^—As(V) 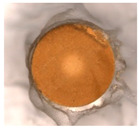	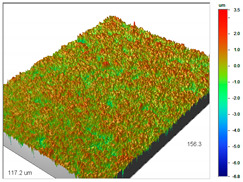	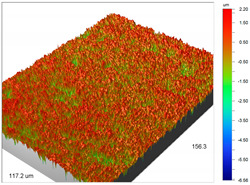
Roughness parameters	R_a_—627.51 nm ± 85.34 nm R_q_—790.80 nm R_t_—10.34 um	R_a_—625.36 nm ± 85.05 nm R_q_—788.23 nm R_t_—7.76 um

**Table 6:** The elemental composition determined for Dowex M 4195.

Name	Position [eV]	Area	% Atom Concentration	% Mass Concentration
C 1s	284.7	42,140.11	82.2	76.2
O 1s	531.6	11,815.51	7.9	9.7
N 1s	399.3	6908.79	7.5	8.1
S 2p	168.3	2069.96	2.4	6.0

**Table 7:** The XPS analysis results obtained in the narrow range of binding energy for the Dowex M 4195.

Name	Position [eV]	Area	% Atom Concentration	Phase
O 1s	531.4	812.25	68.9	SO_4_^2−^, C=O
O 1s	532.9	365.79	31.1	O-H
C 1s	284.7	2322.86	66.6	C=C, C-C, C-H
C 1s	286.0	868.44	24.9	C-N
C 1s	287.5	82.66	2.4	C=O, C-N
C 1s	289.0	35.96	1.0	Shake-up
C 1s	290.8	52.84	1.5	π-π
C 1s	292.3	127.03	3.6	π-π
N 1s	399.3	349.52	70.5	N-pyridine
N 1s	401.7	146.36	29.5	C-N
S 2p 3/2	167.9	120.52	100.0	SO_4_^2−^
S 2p 1/2	169.0	60.26	-	-

**Table 8:** The elemental composition determined for Dowex M 4195 after the sorption of Cu(II).

Name	Position [eV]	Area	% Atom Concentration	% Mass Concentration
C 1s	284.7	43,824.25	78.6	68.7
O 1s	531.3	14,057.58	8.6	10.1
N 1s	399.0	8241.83	8.2	8.4
S 2p	168.0	1794.61	1.9	4.5
Cl 2p	198.0	2417.58	1.9	4.8
Cu 2p	932.1	10,911.83	0.8	3.5

**Table 9:** The results of XPS analysis obtained in a narrow range of binding energy for Dowex M 4195 after Cu (II) sorption.

Name	Position [eV]	Area	% Atom Concentration	Phase
O 1s	531.3	655.87	63.2	SO_4_^2−^, C=O
O 1s	532.9	382.52	36.8	O-H
C 1s	284.7	2054.58	60.6	C=C, C-C, C-H
C 1s	286.0	929.85	27.4	C-N
C 1s	287.4	119.93	3.5	C=O, C-N
C 1s	289.0	59.05	1.7	Shake-up
C 1s	290.2	36.17	1.1	π-π
C 1s	292.3	191.03	5.7	π-π
N 1s	399.6	465.61	81.9	N-pyridine
N 1s	401.6	102.64	18.1	C-N
S 2p 3/2	167.8	75.16	100.0	SO_4_^2−^
S 2p 1/2	169.0	37.58	-	-
Cl 2p 3/2	197.8	97.94	100.0	Cl^−^
Cl 2p 1/2	199.4	48.97	-	-
Cu 2p 3/2	932.6	520.86	74.9	Cu(0). Cu(I)
Cu 2p 1/2	952.5	260.43	-	-
Cu 2p 3/2	935.0	173.88	25.1	Cu(II)
Cu 2p 1/2	955.2	86.94	-	-

## Data Availability

The data presented in this study are available on request from the corresponding author.
